# Assessment and referral of patients with short stature by primary care physicians in the Arabian gulf region: Current perspectives from a regional survey

**DOI:** 10.3389/fped.2022.988614

**Published:** 2022-11-25

**Authors:** W. Kaplan, E. Al Amiri, N. Attia, I. Al Basiri, I. Romany, E. Al Shehri, A. Al Twaim, S. Al Yaarubi, A. Deeb

**Affiliations:** ^1^ Department of Pediatrics, Tawam Hospital, Al Ain, United Arab Emirates; ^2^ Diabetes and Endocrine Unit, Department of Pediatrics, Al Qasimi Women and Children Hospital, Sharjah, United Arab Emirates; ^3^ Pediatric Endocrine Unit, Department of Pediatrics, King Abdulaziz Medical City and King Abdullah Research Centre, Jeddah, Saudi Arabia; ^4^ Diabetic and Endocrine Unit, Department of Pediatrics, Mubarak Al Kabeer Hospital, Kuwait City, Kuwait; ^5^ Department of Medical Affairs, Pfizer Gulf FZ LLC, Dubai, United Arab Emirates; ^6^ Department of Pediatrics, International Diabetes Care Center, Jeddah, Saudi Arabia; ^7^ Pediatric Endocrine Division, Department of Pediatrics, Prince Sultan Military Medical City, Riyadh, Saudi Arabia; ^8^ Department of Pediatrics, Oman Medical Specialty Board, College of Medicine and Health Science, Muscat, Oman; ^9^ Division of Paediatric Endocrinology, Sheikh Shakhbout Medical City, Abu Dhabi, United Arab Emirates; ^10^ College of Medicine and Health Sciences, Khalifa University, Abu Dhabi, United Arab Emirates

**Keywords:** children, short stature, Arabian Gulf region, growth hormone therapy, survey

## Abstract

Children with short stature are frequently referred late to pediatric endocrinologists in the Arabian Gulf region. This is likely a contributing factor to late initiation of treatment despite current evidence suggesting that children with short stature have better outcomes with earlier treatment. This delay in referral could be due to a lack of identification or proper assessment of short stature by front-line physicians. To analyze the assessment and perception of short stature in this group of physicians, an expert group of pediatric endocrinologists developed and disseminated an anonymous online survey of 22 multiple choice questions amongst general pediatricians, pediatric subspecialists, and family medicine physicians in the Arabian Gulf region. Of the 640 respondents, 450 completed the survey (70.3% completion rate). While most surveyed physicians use the correct definition for short stature in children, only 24% reported a consistent use of a wall-mounted stadiometer. Of the respondents, 50% or less would consider referring clinical conditions other than growth hormone (GH) deficiency or idiopathic short stature, 41% would refer a child with short stature as soon as height dropped below the 5th percentile, 57% considered GH a treatment option for short stature, and only 60% consider GH treatment safe. The results of this survey demonstrate knowledge gaps in short stature assessment and referral that need to be addressed through education on short stature amongst target physicians, and lay groundwork for future recommendations to address those gaps in the Arabian Gulf region.

## Introduction

Growth from fetus to adolescence is the product of age- and gender-dependent interactions with genetic, environmental, lifestyle and physiological factors (1).

Growth within the wide range of normative patterns is an indication of good general health (1), and monitoring growth is considered fundamental in evaluating child's health (2). Growth that is markedly lower than the normative range may lead to short stature (1) which may compromise the physical and psychological health of a child (2).

Early detection of abnormal growth in childhood allows for timely intervention that may prevent excessive short stature in adulthood (2, 3). Many studies of growth hormone (GH) therapy for different conditions showed that early initiation of treatment increases the likelihood of achieving the genetic height potential (4–7).

Child healthcare providers at different levels contribute to the assessment and management of children with growth disorders. This includes routine screening of height in primary care, establishing a differential diagnosis for short stature, early identification and referral for eligible conditions, and consideration of therapeutic options by pediatric endocrinologists at tertiary care (8).

Short stature is one of the most common reasons for referrals to pediatric endocrinologists (3, 9). However, a retrospective review of children with short stature referred to a pediatric endocrinologist at an endocrine clinic in Kuwait found that approximately one-fifth of referred children had normal growth (9). This observation suggests that there is a need to improve the referral process so that unnecessary investigations and parental anxiety are avoided.

Conversely, despite studies suggesting better outcomes with earlier intervention (4–7), there is evidence that many children start GH therapy later than desired (10–12). Delayed referral is likely to be a major contributing factor for the late initiation of treatment.

The aim of this study was to evaluate the knowledge and perception of short stature among pediatricians, pediatric sub-specialists, and family medicine specialists in the Arabian Gulf region.

## Materials and methods

An expert group comprising pediatric endocrinologists from the Arabian Gulf region, selected based on their expertise in short stature, was formed and first convened in May 2021. The group hypothesized that delayed referral to pediatric endocrinologists could be secondary to one or more of the following reasons: (1) mis-diagnosis due to using wrong definition, invalid growth chart, or inaccurate height measurement; (2) Misperception by the primary healthcare provider or families about normal growth, or the efficacy and safety of GH; (3) Lack of knowledge about the full approved indications of GH; and (4) Limited approval for the treatment with GH. To investigate the above possibilities, an anonymous online survey was developed, validated by the expert group, and disseminated (13). The survey was composed of 22 multiple choice questions and focused on the assessment and diagnosis of short stature, indication and time of referral, safety and efficacy of GH, and the status of GH treatment coverage. Additionally, the group added a question to assess the pre-referral work up. A link to the survey was circulated by email through the expert group members to general pediatricians and family medicine physicians in their institutions and forwarded to the national and regional societies of general pediatricians and family medicine physicians in their countries. The survey was kept open for one month.

## Survey results

Of the 640 respondents, 450 completed the survey (70.3% completion rate). Respondents were from Saudi Arabia (37.8%), United Arab Emirates (25.6%), Kuwait (25.0%), Oman (6.8%), Qatar (3.2%), and Bahrain (1.5%). The respondents were general pediatricians (36.4%), pediatric sub-specialists (25.1%), family medicine physicians (14.7%), general practitioners (11.6%) and general pediatricians with an interest in endocrinology (5.1%), 7.1% selected “Other”. Of the respondents, 75.6% reported practicing in government facilities, 15.5% in private practice, and 8.9% combined government/private healthcare facilities. The majority (77.5%) worked as part of a multispecialty team with the remainder (22.5%) operating as sole practitioners. Most of the respondents (77.5%) had more than 10 years of clinical experience, and 40.9% had an experience of more than 20 years.

### Definition of short stature

**Figure 1:**
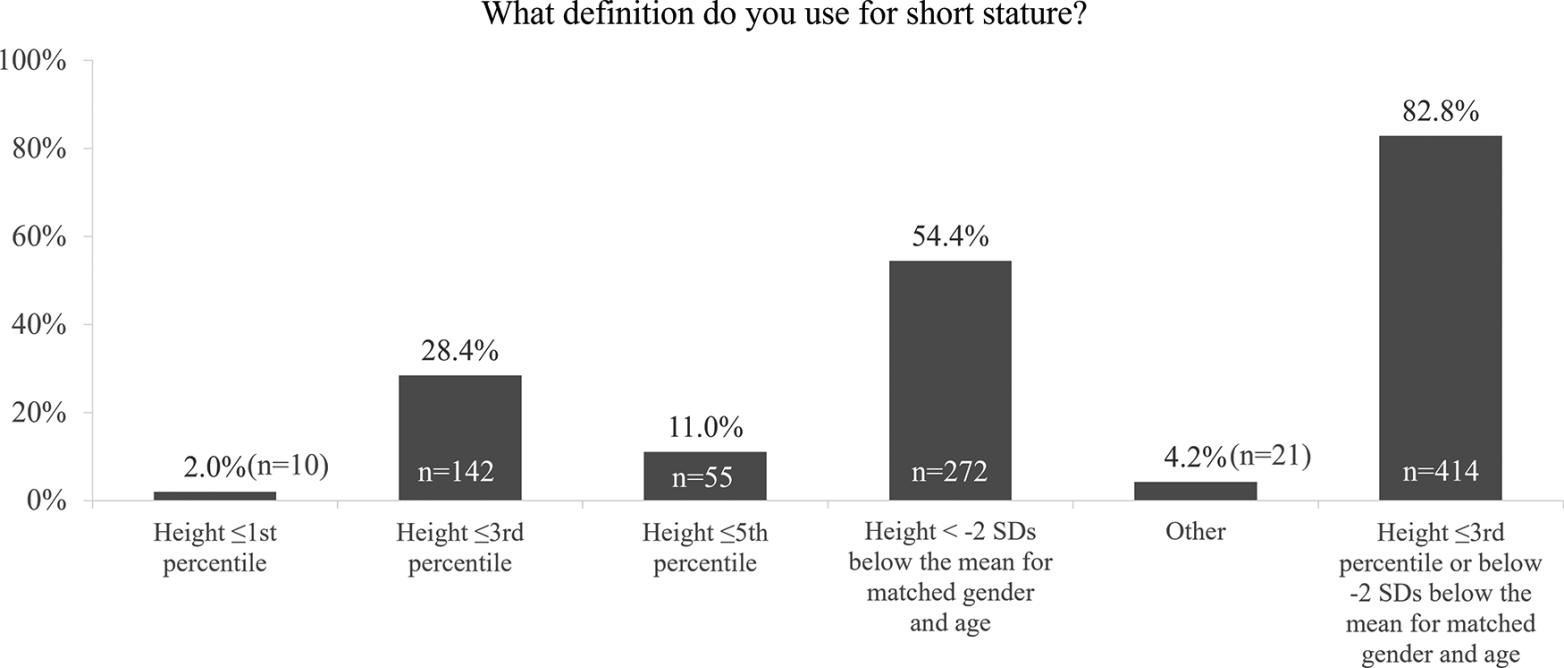
Survey response “What definition do you use for short stature?”. SD, standard deviation.

The survey found that the majority (>80%) of respondents used the correct definition of short stature, i.e., height less than two standard deviation score (2 SDS) below the mean for matched gender and age, or height less than the 3rd percentile (Figure 1) (14).

### Screening and assessment of short stature

**Figure 2:**
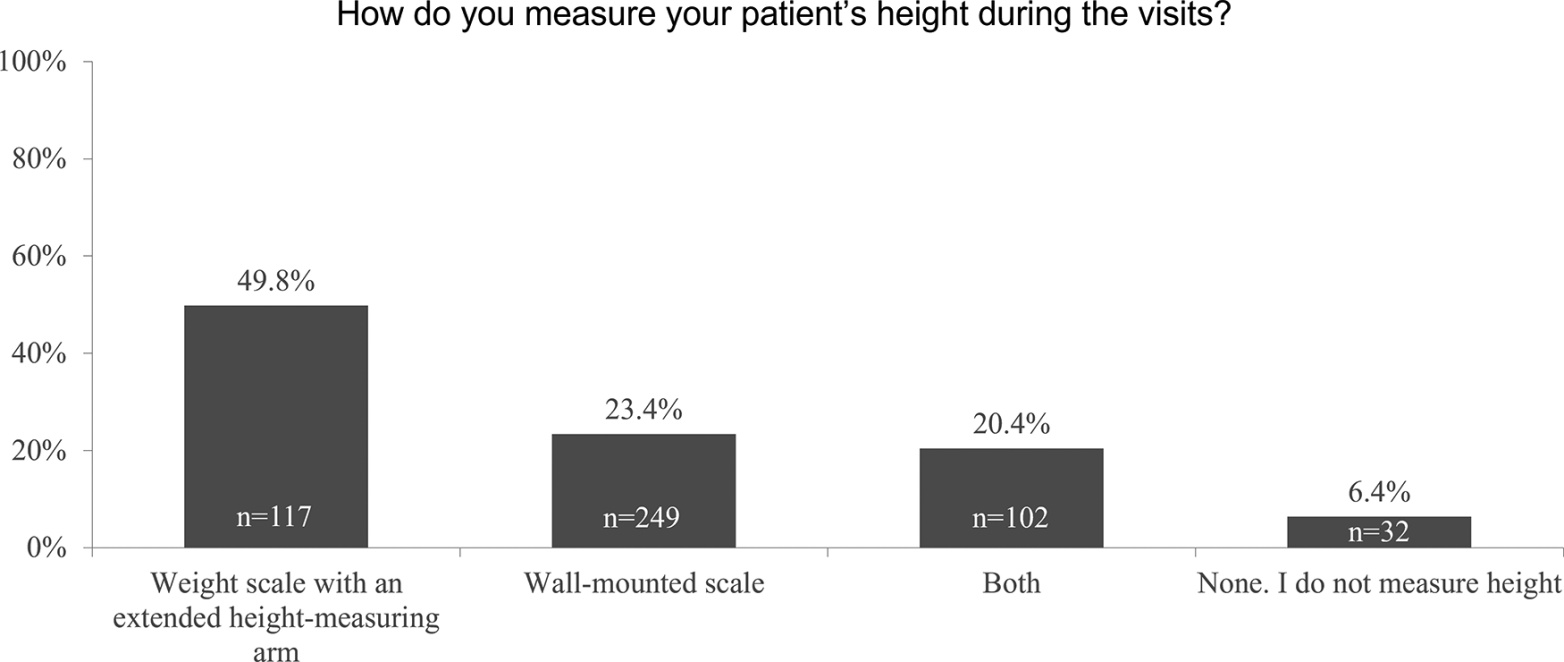
Survey response “How do you measure your patient's height?”.

While more than 80% of respondents use approved national or international (predominantly the World Health Organization [WHO] and Centers for Disease Control and Prevention [CDC]) growth charts, fewer than 25% reported measuring their patients' height by using the wall-mounted stadiometer exclusively (Figure 2).

**Figure 3:**
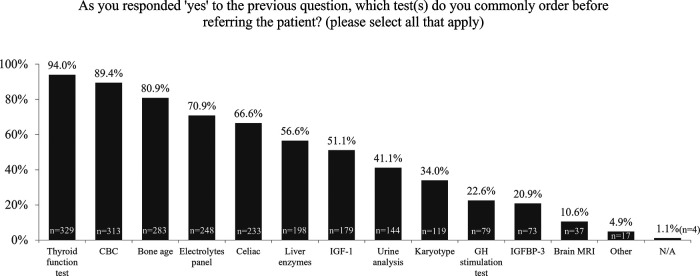
Survey responses to “Which test/s do you commonly order before referral?”. CBC, complete blood count; GH, growth hormone; IGF, insulin-like growth factor; IGFBP, insulin-like growth factor binding protein; MRI, magnetic resonance imaging; N/A, not applicable.

A majority (71.4%) of the respondents would initiate work-up for short stature before referring the patient. The most common reported tests prior to referral were thyroid function, complete blood count, and bone age, while only 51.1% of respondents indicated they would check insulin-like growth factor 1 (IGF-1) prior to referral (Figure 3).

### Age at referral of children with short stature

A minority of the respondents (6.1%, and <5%) indicated they would refer patients younger than 2 years or after the onset of puberty, respectively, while 24.5% and 24.1% reported that the ideal age for referral is 2–5 years and 5–10 years, respectively. The majority (40.8%) responded that a child with short stature should be referred to a pediatric endocrinologist as soon as their height drops below the 5th percentile.

### Perception of growth hormone therapy for short stature

The majority of the respondents (61.3%) indicated that caregivers would be in favor of initiating GH treatment for their children. While 36.6% highlighted that caregivers would be hesitant to initiate GH therapy and 2.1% selected that caregivers would be against initiating GH.

**Figure 4:**
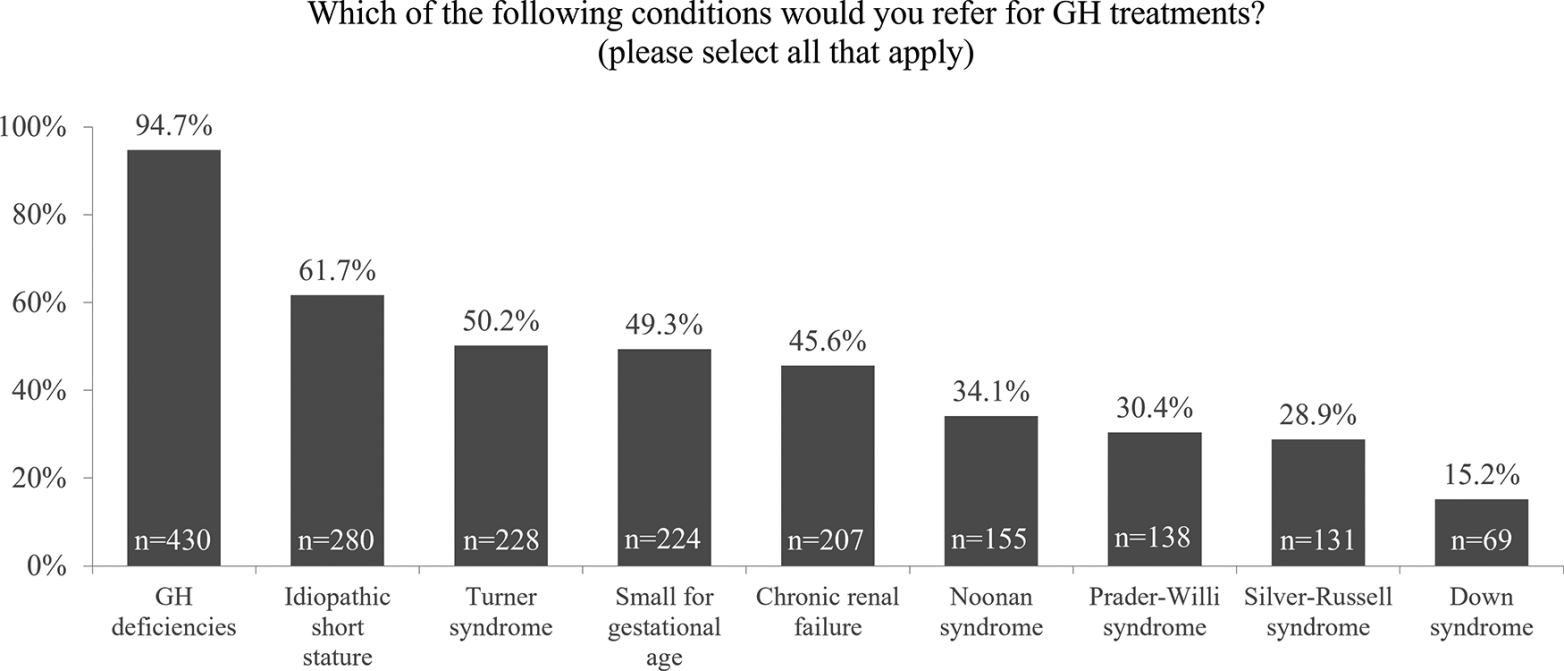
Survey responses to “Which of the following conditions would you refer for GH treatment?”. GH, growth hormone.

From the healthcare providers' perspective, GH deficiency was selected as the most common condition for referral for GH therapy followed by idiopathic short stature (ISS) (Figure 4). However, 50% or less of survey respondents would refer patients with other approved conditions for GH therapy.

Around 56% of respondents consider GH therapy an option for children with short stature, and only 60% indicated that GH treatment is safe for children.

**Figure 5:**
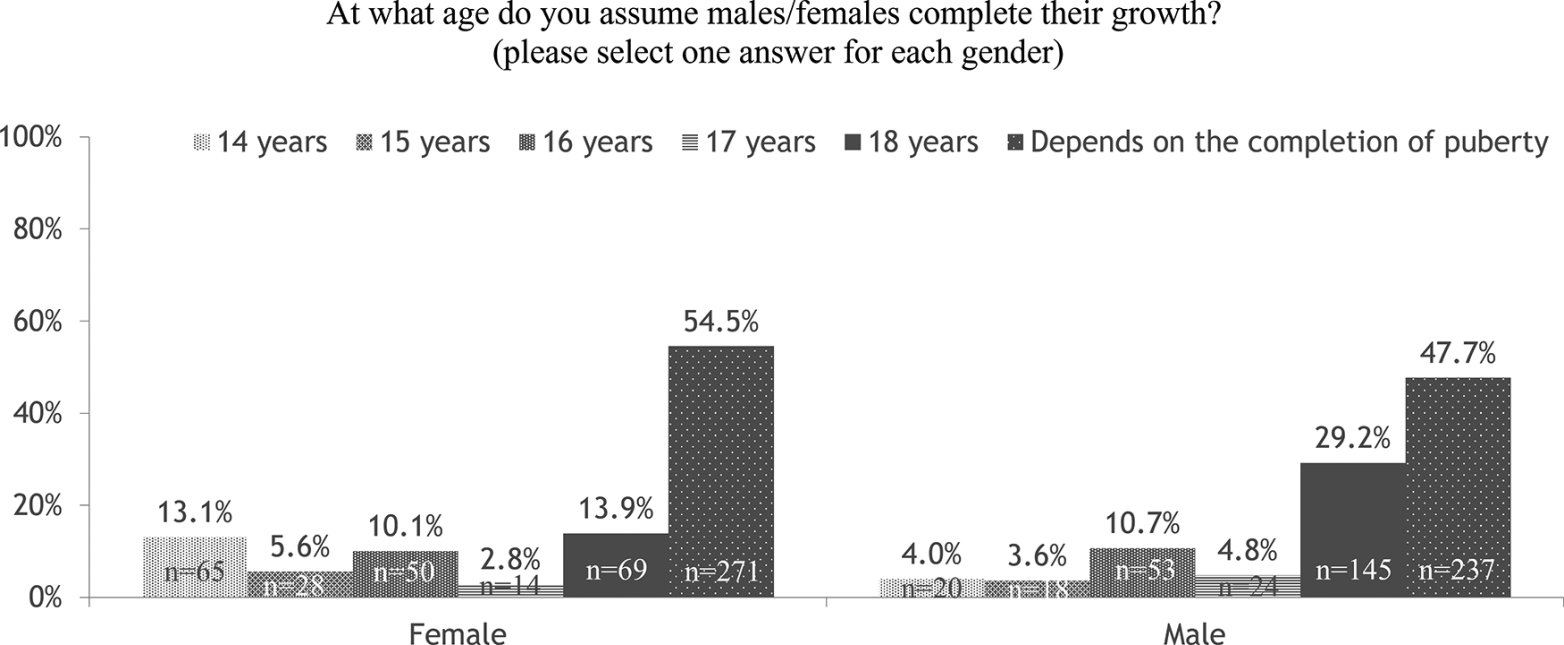
Survey responses to “At what age do you assume males/females complete their growth?”. GH, growth hormone.

Only 47.7% and 54.5% of respondents assumed the age at completion of growth correlates with completion of puberty for males and females, respectively (Figure 5).

Of the respondents, only 51% reported GH treatment coverage by either government or private insurance payors.

## Discussion

Results from our survey and other studies conducted within the Arabian Gulf region (8, 9) highlight challenges and knowledge gaps in the identification and management of children with short stature.

Growth is a continuous, rather than linear, physiological process comprising infantile, childhood, and pubertal phases (3). Accurate measurement is the first step in the evaluation of a child with suspected short stature (15). A horizontal ruler should be used to measure length in children younger than two years, and a wall-mounted stadiometer for children older than two years (15). The survey result identified a major gap where less than 25% of respondents were complying with these recommendations.

As per the international and regional recommendations in the Arabian Gulf, the WHO growth charts are recommended for children up to 2 years of age, and the CDC growth charts can be used for older children (8, 16). However, local validated growth charts, when available, such as in the Saudi Arabia, are preferred as they better reflect the effects of genetic, ethnic, nutritional, and environmental factors on growth (17). This was reported when comparing the 2000 CDC growth charts with the Saudi growth charts in Saudi children and adolescents (18), and also when comparing the Finnish population-specific growth charts with the 2006 WHO charts in screening for Turner syndrome (19). The result of our survey shows that the vast majority of the respondents are compliant with the above recommendations.

Investigation of IGF-1 is frequently indicated in the assessment of short stature, and because of its minimal circadian variation, a single measurement is more reliable than a single basal GH value (20). Only 51% of the survey respondents indicated ordering IGF-1 as part of the laboratory evaluation of short stature. This was attributed, partially, to restricting the test ordering to pediatric endocrinologists. We recommend that primary care physicians should have access to ordering IGF-1 when evaluating children with short stature. However, this test limitation in young children and those with abnormally low body mass index (BMI) should be taken into consideration (8). Additionally, due to the effect of age, gender, BMI, and pubertal status on the levels of IGF-1 (20, 21), it is preferred that the result should be expressed as SDS against age, gender and Tanner stage-matching reference ranges (21).

Evidence supporting the safety profile of GH therapy includes large registries comprising more than 600,000 patients-years of GH exposure, and long-term safety cohorts of adults who received GH therapy as children (22). Several studies have reported favorable outcomes of GH treatment in the Arabian Gulf region (23–26). Despite this, only 60% of the respondents considered GH safe. Additionally, less than half of the survey respondents demonstrated knowledge about the approved indications of GH beyond GH deficiency and ISS. Most respondents (>75%) expressed interest in learning more about the etiology, assessment, and management of short stature.

Ideally, children with abnormal short stature should be referred for further diagnosis and for treatment, if indicated (27). Algorithms and referral criteria have been developed to facilitate identification of the causes of short stature based on body proportions, growth velocity, and mid-parental height, and to support child healthcare professionals in their decision to either refer a child with short stature or monitor their growth (28). Data from our survey reflect that referral patterns may be impacted by differences in healthcare structures within and between countries across the Arabian Gulf region, particularly in terms of government-based and private-based payment approval for diagnostic testing and treatment. The expert group will continue its efforts highlighting the long-term benefits of GH treatment when prescribed for the approved indications, in an effort to convince the decision-makers in the region to expand the list of covered medical conditions.

### Conclusions and future endeavors

Our survey demonstrates that there are gaps in the assessment of children with short stature, uncertainty about appropriate referral of these children to pediatric endocrinologists, and a lack of understanding of the safety and approved indications for GH treatments. Future local and regional educational programs that address these areas should be provided physically and virtually by the local experts in each country in partnership with societies whose members are referring physicians. It is also important to ensure that updated international and regional guidelines are widely available and accessible. Addressing these knowledge gaps should form the basis for future recommendations and plans in the region.

## Data Availability

The original contributions presented in the study are included in the article/Supplementary Material, further inquiries can be directed to the corresponding author.
